# A Graph-Based Algorithm for Computing Matrix Elements of Arbitrary Operators between Configuration State Functions

**DOI:** 10.1021/acs.jpca.5c08310

**Published:** 2026-02-18

**Authors:** Ignacio Fdez. Galván, Mitra Rooein, Roland Lindh

**Affiliations:** † Department of Chemistry for Life Sciences, 8097 Uppsala University, P.O. Box 576, 75123 Uppsala, Sweden; ‡ Uppsala Center for Computational Chemistry (UC_3_), Uppsala University, P.O. Box 576, 75123 Uppsala, Sweden

## Abstract

We present a graph-based algorithm for computing matrix elements of arbitrary second-quantized operators between configuration state functions (CSFs) defined in a genealogical scheme. Unlike Slater determinants, CSFs are spin-adapted and offer a more compact representation of many-electron wave functions, but their use in quantum chemical methods is often hindered by the complexity of evaluating matrix elements. Our approach leverages a graphical representation to efficiently encode the expansion of CSFs in terms of Slater determinants without explicitly constructing the full expansion. The algorithm applies operator sequences directly to the graph and computes overlaps via graph traversal, yielding matrix elements, and is completely general for any operator sequence. Numerical tests demonstrate that the method achieves machine-level precision and outperforms explicit determinant expansion by several orders of magnitude. This framework opens new possibilities for CSF-based implementations in selected and stochastic configuration interaction methods.

## Graphical abstract

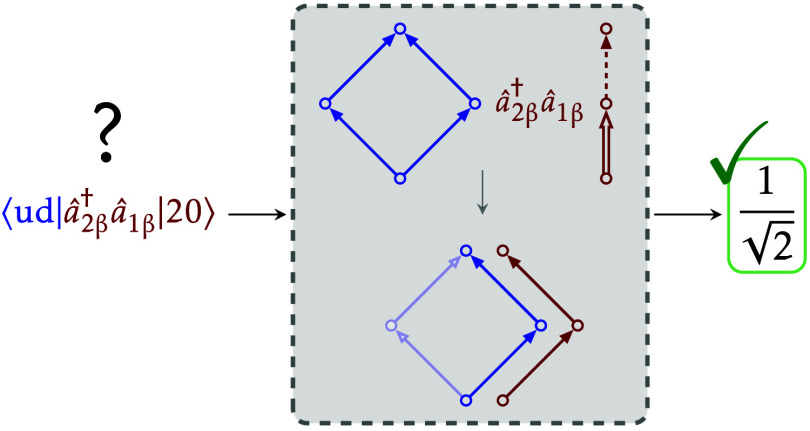

## Introduction

Slater determinants are the fundamental antisymmetric many-electron basis of most quantum chemical methods. They provide a conceptual simplicity and very efficient ways to compute matrix elements over any operator via the Slater–Condon rules. However, Slater determinants are not eigenfunctions of the total spin operator *Ŝ* ^2^ (except in some trivial cases), which introduces spin contamination, particularly problematic in open-shell systems or excited states.

Configuration state functions (CSFs)^1^ are by construction antisymmetrized eigenfunctions of *Ŝ* ^2^ and thus avoid spin contamination while simultaneously reducing the number of parameters in CI expansion. Additionally, the use of CSFs allows for some other compression techniques that can even further decrease the effective CI size and increase sparsity.^2^ The main drawback is that the calculation of coupling coefficients and sigma vectors in CSF basis is not as trivial as in Slater determinant basis, and many quantum chemistry codes that employ CSFs actually perform a transformation to Slater determinants for some of these calculations.^3^ The most common framework for computing the needed quantities in a CSF basis is the unitary group approach (UGA), notably developed by Paldus et al.^4–6^ For the specific case of CSFs defined in a so-called genealogical scheme and singlet one- and two-electron excitation operators, the graphical unitary group approach (GUGA) furnishes very efficient recipes,^7,8^ while a similar formalism allows the treatment of spin-dependent operators.^9–11^ But there is a continuing effort to find alternative ways for defining CSFs and computing the necessary matrix elements.^12–17^

In this work we propose a simple graphical method to obtain matrix elements of arbitrary operators, expressed as sequence of elementary second-quantization operators, over genealogical CSFs, and present an explicit algorithm. It is our expectation that this method can serve as a basis for even more compact formulas for specific operators, thus extending the applicability of purely CSF-based methods without resorting to transformation to Slater determinants.

## CSFs and Slater Determinants

Given an ordered set of *n*_o_ orthonormal orbitals (also called levels), a genealogical CSF can be represented as a list or vector ***t***, of length *n*_o_, each element taking the values 0, 1, 2, or 3 in the GUGA convention.^7^ In this work we will use a more intuitive labeling and the values will be, respectively 0 (empty), u (up), d (down), 2 (doubly occupied). Each *t*_*i*_ element specifies the occupation of orbital *i* (occ­(*t*_*i*_) ∈ {0, 1, 2}) and the change in cumulative spin introduced by this orbital ( $\Delta S\left(t_{i}\right)\in \left\{0,\pm \frac{1}{2}\right\}$ ), as collected in Table 1. In this way, the intermediate number of electrons and spin at each level can be defined respectively as *N*_*i*_ = ∑_*k* = 1_^*i*^ occ­(*t*_*k*_) and *S*_*i*_ = ∑_*k* = 1_^*i*^ Δ*S*(*t*_*k*_), and the total number of electrons and spin are *N* = *N*_*n*_o__ and *S* = *S*_*n*_o__. Valid CSFs require that all *S*_*i*_ be nonnegative, and to fully define a CSF an additional spin projection value, *M*, must be specified, such that *M* ∈ {−*S*, −*S* + 1,..., *S* – 1,*S*}. Thus, in bra–ket notation the CSF will be written as | ***t***, *M*⟩.

**Table 1:** Equivalence between the Step Values Used in GUGA and the Labels Used in This Work, Together with the Occupation and Change in Spin Corresponding to Each Case

*t*_*i*_			
GUGA step case	label in this work	occ(*t*_*i*_)	Δ*S*(*t*_*i*_)
0	0	0	0
1	u	1	$+\frac{1}{2}$
2	d	1	$-\frac{1}{2}$
3	2	2	0

Similarly, a Slater determinant can be represented by a list or vector ***p***, of length *n*_o_ as well, each element of which takes the values 0, α, β, or 2. In this case the *p*_*i*_ values define the occupation and the change in cumulative spin *projection*, as indicated in Table 2. Intermediate and total number of electrons can be obtained as for a CSF (using *p*_*i*_ instead of *t*_*i*_), and intermediate and total spin projections are defined as *M*_*i*_ = ∑_*k* = 1_^*i*^ Δ*M*(*p*_*k*_) and M = *M*_*n*_o__.

**Table 2:** Occupation and Change in Spin Projection for the Different Element Values of a Determinant

*p*_*i*_	occ(*p*_*i*_)	Δ*M*(*p*_*i*_)
0	0	0
α	1	$+\frac{1}{2}$
β	1	$-\frac{1}{2}$
2	2	0

A CSF can be expanded as a linear combination of Slater determinants

1 $$\left|t,M\right\rangle =\sum_{k}c_{k}\left|p^{\left(k\right)}\right\rangle$$

where the sum is restricted to all determinants that have the same occupation pattern as ***t*** (occ­(*p*_*i*_) = occ­(*t*_*i*_)), and the same spin projection *M*. All values related to spin, *S*, *M*, *S*_*i*_, etc., take integer or half-integer values. For convenience, we will mostly use the corresponding doubles, *b* = 2*S* and *x* = 2*M* (and similarly for *b*_*i*_, etc.), which are always integers. For a genealogical CSF, the expansion coefficients *c*_*k*_ are easily obtained from ***t*** and ***p*** ^(*k*)^ ^18^

2 $$c_{k}=\left\langle p^{\left(k\right)}\right|t,M\rangle =\prod_{i=1}^{n_{o}}C_{t_{i},p_{i}^{\left(k\right)}}\left(b_{i},x_{i}^{\left(k\right)}\right)$$

where the *C*’s are derived from Clebsch–Gordan coefficients. We use a form of these factors that is consistent with the UGA phase convention^19–21^

3 $$\begin{array}{ll} C_{0,0}\left(b,x\right)=1 & C_{2,2}\left(b,x\right)={\left(-1\right)}^{b}\\ C_{u,\alpha }\left(b,x\right)=\sqrt{\frac{b+x}{2b}} & C_{d,\alpha }\left(b,x\right)={\left(-1\right)}^{b+1}\sqrt{\frac{b-x+2}{2\left(b+2\right)}}\\ C_{u,\beta }\left(b,x\right)=\sqrt{\frac{b-x}{2b}} & C_{d,\beta }\left(b,x\right)={\left(-1\right)}^{b}\sqrt{\frac{b+x+2}{2\left(b+2\right)}} \end{array}$$

## Graphical Representation of CSFs

The determinant expansion of a genealogical CSF can be represented in a two-dimensional lattice directed acyclic graph. Starting from a root node at coordinates (0,0), each orbital adds edges connecting the nodes at level *i* – 1 with nodes at level *i*. For *t*_*i*_ ∈ {0, 2}, the edges are vertical, connecting each (*x*, *i* – 1) with (*x*, *i*). To distinguish the two cases, the edges are assigned a different type, or drawn in different style, representing *p*_*i*_ = 0 and *p*_*i*_ = 2. For *t*_*i*_ ∈ {u, d}, the edges join (*x*, *i* – 1) with (*x* + 1,*i*), representing *p*_*i*_ = α, and with (*x* – 1, *i*), representing *p*_*i*_ = β. Each edge is assigned a value or weight according to eq 3, for which *p*_*i*_ is taken from the edge type, and *x* is the horizontal coordinate of the target node of the edge. Edges with a zero weight can be removed. As a consequence, only nodes with *x* ∈ {−*b*_*i*_, −*b*_*i*_ + 2,..., *b*_*i*_ – 2, *b*_*i*_} need to be considered, i.e., the intermediate *M*_*i*_ values must be consistent with the intermediate *S*_*i*_ values. Graphically, the sequence of *b*_*i*_ values defines a boundary such that all the graph edges must be contained in the enclosed area.^1^

Each path starting at the root node and ending an any (*x*, *i*) node represents a determinant with *i* orbitals, with occupations matching those of ***t*** up to level *i*, and with $M=\frac{1}{2}x$ . Thus, the CSF | ***t***, *M*⟩ is a linear combination of all determinants represented by paths ending at the node (2*M*, *n*_o_). The coefficient of each determinant in this linear combination is the product of the edge weights in its path. An example is shown in Figure 1, from which it is quickly obtained that the coefficient of the determinant |*αβα*0*αβ*2α⟩ in the expansion of |uud0uu2d, M = 1⟩ is 1 × $\sqrt{\frac{1}{2}}\times \sqrt{\frac{1}{3}}$ × 1 × 1 × $\sqrt{\frac{1}{3}}$ × (−1) × $\left(-\sqrt{\frac{1}{4}}\right)$ = $\sqrt{\frac{1}{72}}$ .

**Figure 1:**
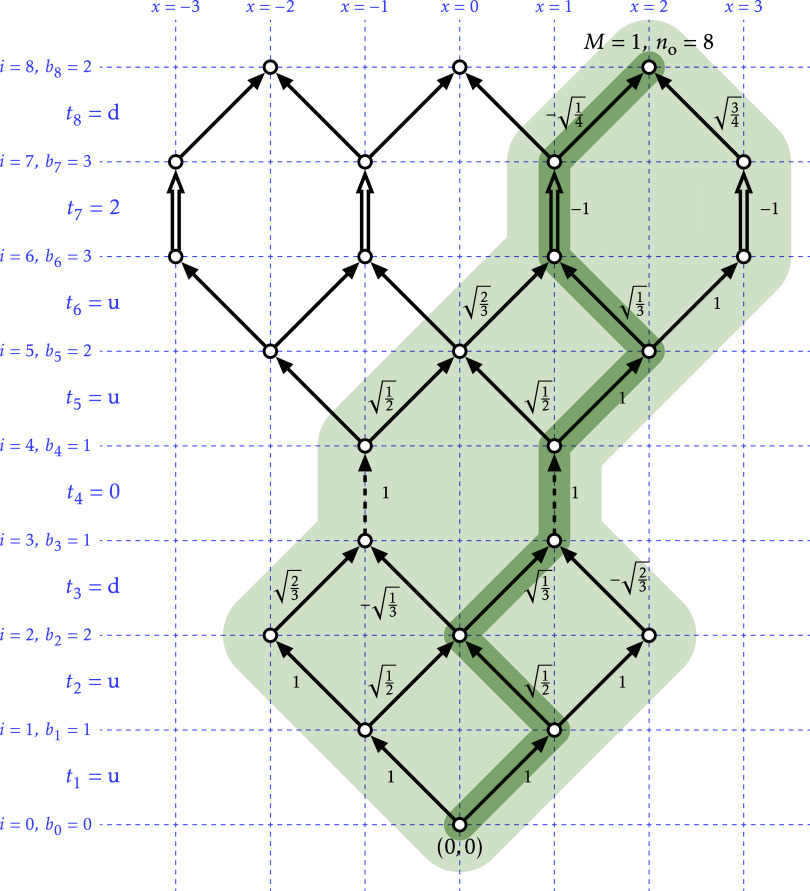
Graphical representation of the CSF |uud0uu2d, *M* = 1⟩. The shaded area contains all determinant paths that enter the CSF linear combination, ending at the node (2,8). The other two nodes at level *i* = 8 are the end points for CSFs with the same ***t*** vector but different *M* values. The numbers next to each edge are the edge weights. Note that edges for *p*_*i*_ = *t*_*i*_ = 0 are drawn with dashed lines, and edges for *p*_*i*_ = *t*_*i*_ = 2 are drawn with double lines. In a darker shade, the path representing the determinant |*αβα*0*αβ*2α⟩.

This representation, which we will call genealogical determinant graph (GDG), allows other linear combinations of determinants which are not CSFs, as long as the coefficient for each determinant is equal to the product of the edge weights. Every genealogical CSF can be represented as a GDG, but not every GDG corresponds to a CSF. An arbitrary GDG ending at a single node has a well-defined *M* value (given by *x*/2), but not a well-defined spin or, unless each level contains edges with the same occupation, orbital occupation. This GDG representation is essentially a form of graphically contracted function (GCF), introduced by Shepard et al.^22–24^ to express linear combinations of CSFs, and already used by Fitzpatrick^25^ to compactly describe the composition of a CSF in terms of Slater determinants; we extend this usage by including empty and doubly occupied orbitals, and explicitly allowing other linear combinations of Slater determinants that are not CSFs.

## Graph Overlaps

The overlap between two GDGs can be computed by first overlaying the two graphs, discarding the edges that are not present in both, or that have different types, and assigning to the surviving edges the product of the weight in the two graphs. Then a “norm” is computed for this “overlap graph”, by initially assigning a value 1 to the root node at (0,0) and then, to each other node, the sum of the values of the nodes at the previous level connected to it, multiplied each of them by the weight of the edge that connects the two nodes. The final result for the overlap is the sum of the values of the end nodes (the nodes that have no edge connecting it to a higher level). In the case of CSFs, there will be a single end node. This process is equivalent to the way wave function overlaps are computed in the GCF method.^22^

The orthonormality of CSFs is easy to prove. The norm of a CSF is the square root of the overlap with itself. The overlap graph of a CSF with itself is just the same graph as for the CSF, but with all edge weights squared. From eq 3 the following identities are obvious, for any *b* and *x*

4 $$C_{0,0}{\left(b,x\right)}^{2}=C_{2,2}{\left(b,x\right)}^{2}=C_{u,\alpha }{\left(b,x\right)}^{2}+C_{u,\beta }{\left(b,x\right)}^{2}=C_{d,\alpha }{\left(b,x\right)}^{2}+C_{d,\beta }{\left(b,x\right)}^{2}=1$$

so the sum of the edge weights arriving to any node is always 1. However, each weight should be multiplied by the node value it connects from. But if at any level *i* – 1 all node values are equal, then all node values at level *i* will be equal to that same value too. Since the node value at the root is 1, all the node values at every other level will also be 1, and as there is a single end node, the final norm is 1 and any CSF is normalized.

In a similar manner, the overlap between two CSFs can only be nonzero if they have the same end node (have the same *n*_o_ and *M*). They also must have the same occupation pattern, otherwise at the level where the occupations differ there will be no matching edges. The only difference then could be in levels which are of u type in one CSF and of d type in the other. At the first level with such difference, the *b*_*i*_ values will differ by 2 (the *b*_*i*–1_ values are equal, for one CSF it increases by 1, for the other it decreases by 1). But in that case one would have, for any *b* and *x*

5 $$C_{u,\alpha }\left(b+2,x\right)C_{d,\alpha }\left(b,x\right)+C_{u,\beta }\left(b+2,x\right)C_{d,\beta }\left(b,x\right)=0$$

and with the same reasoning as above, if all node values at *i* – 1 are equal, then all node values at level *i* will be 0 and the final value will also be 0. As it is implied that all levels below this first difference are equal, it will be the case that all node values at level *i* – 1 are 1, and the overlap between any two different CSFs is 0, i.e., they are orthogonal.

## Operations on GDGs

An elementary operator is a single annihilation (*â*_*i*σ_) or creation (*â*_*i*σ_^†^) operator acting on orbital *i* ∈ {1,..., *n*_o_} with spin σ ∈ {α,β}. The effect of an elementary operator on a GDG is straightforward: it only modifies the edges at level *i*, and shifts the *x* coordinates of the nodes at level *i* and above. The edges at level *i* can change type (from slanted to vertical, or vice versa) and their weights can be multiplied by a phase factor of ± 1, or by 0, in which case the edge is removed. These effects are listed in Table 3. Apart from the phase factor and the possible removal, the edge weights remain unchanged. If all the edges at level *i* are removed, the result is simply the null state, otherwise the result of an operator on a GDG is another GDG.

**Table 3:** Effect of an Elementary Operator on a GDG^a^

*Ô*	0	α	β	2
*â*_*i*α_	—	0; (−1)^*N*_*i*_−1^; −1	—	β; (−1)^*N*_*i*_^; −1
*â*_*i*β_	—	—	0; (−1)^*N*_*i*_−1^; +1	α;(−1)^*N*_*i*_−1^; +1
*â*_*i*α_^†^	α; (−1)^*N*_*i*_^; +1	—	2; (−1)^*N*_*i*_−1^; +1	—
*â*_*i*β_^†^	β; (−1)^*N*_*i*_^; −1	2; (−1)^*N*_*i*_^; −1	—	—

a For each type of edge at level *i* (column heading), the table lists the new edge type it will be transformed to, the phase factor to multiply the edge weight by, and the shift in the *x* coordinate for all nodes at level *i* and above. The dashes indicates that the edge is removed.

A sequence of *N* ^op^ elementary operators (*ô*_*k*_) can be written as

6 $$Ô=\prod_{k=1}^{N^{\mathrm{op}}}{ô}_{k}={ô}_{1}{ô}_{2}\cdot \cdot \cdot {ô}_{N^{\mathrm{op}}}$$

where each *ô*_*k*_ is either *â*_*i*_*k*_σ_*k*__ or *â*_*i*_*k*_σ_*k*__^†^. The effect of *Ô* acting on a GDG can be found by applying the elementary operators in reverse sequence, from *ô*_*N* ^op^_ to *ô*_1_. The matrix element of *Ô* between two CSFs, ⟨ ***t*** ′, *M*′|*Ô*| ***t***, *M*⟩, is then the overlap between the GDG representing | ***t*** ′, *M*′⟩ and the GDG representing *Ô*| ***t***, *M*⟩.

As an example, Figure 2 shows the effect of the sequence *â*_1β_^†^ *â*_7β_ on the CSF of Figure 1. First *â*_7β_ acts on level 7, which is of type 2. Table 3 gives α; (−1)^*N*_*i*_−1^; +1 for this case, and according to this the double arrows are replaced with single arrows slanted to the right (α), the edge weights are not modified (*N*_7_ = 7), and the nodes above are shifted to the right (+1). Then *â*_1β_^†^ acts on level 1, which has edges of type α and β. According to Table 3, the β edge is removed, and the α edge is transformed to a vertical double arrow (2), its weight is multiplied by −1 (*N*_1_ = 1), and all nodes above are shifted to the left (−1). Due to the removal of the β edge, two nodes and three edges become disconnected with the root node and can be removed as well.

**Figure 2:**
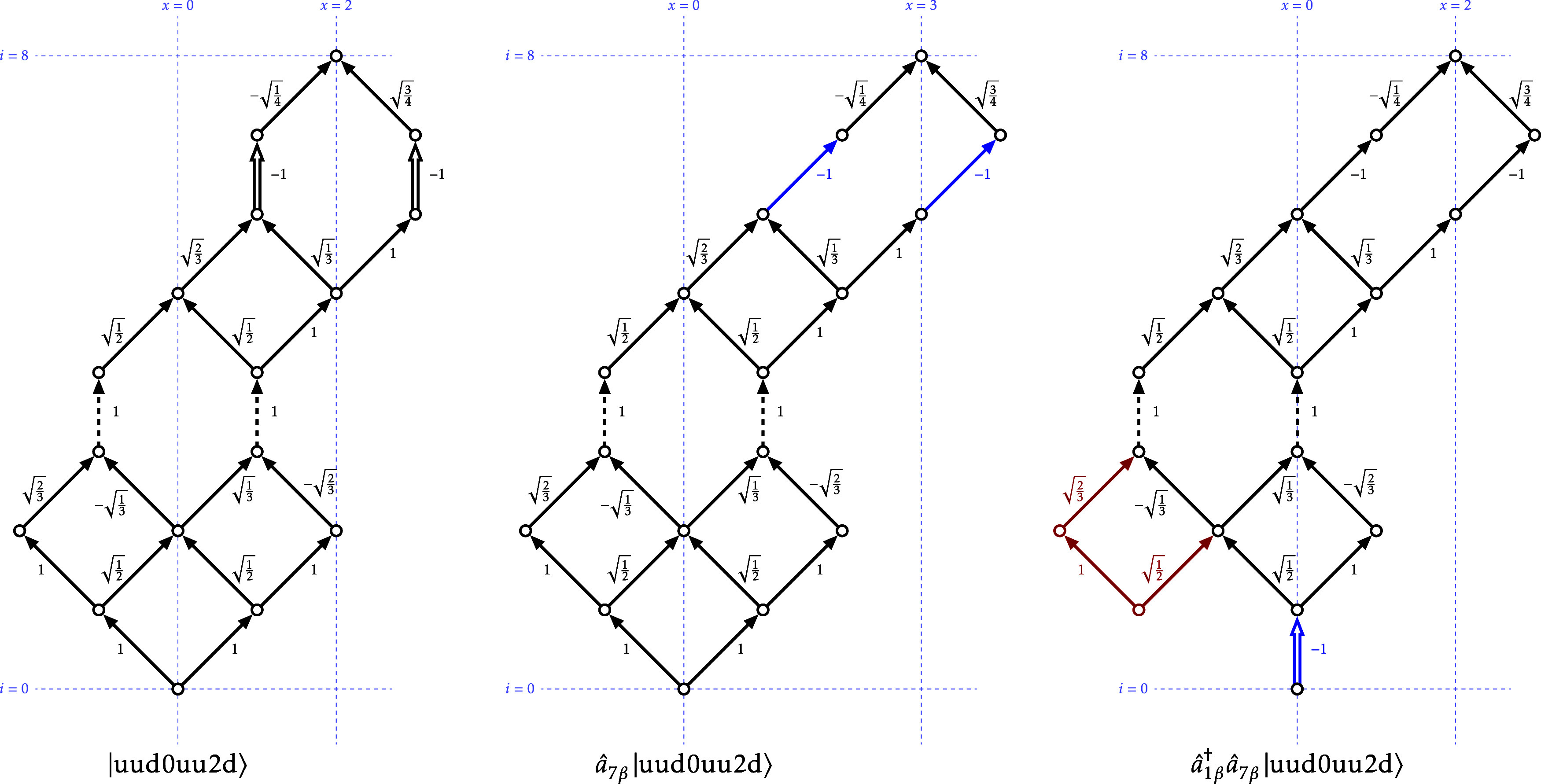
Action of *â*_1β_^†^ *â*_7β_ on the CSF |uud0uu2d, *M* = 1⟩. Left: GDG representing the original CSF. Center: effect of the rightmost elementary operator on the CSF. Right: Effect of the leftmost elementary operator on the middle GDG. The edges modified at each step are drawn in blue. The edges that can be removed are drawn in red.

## Spin Selection Rules

The spin projection of *Ô* is well-defined as *M* ^op^ = ∑_*k* = 1_^*N* ^op^^Δ*M*(*ô*_*k*_), where Δ*M*(*ô*_*k*_) is $+\frac{1}{2}$ for *ô*_*k*_ ∈ {*â*_*i*β_, *â*_*i*α_^†^} and $-\frac{1}{2}$ for *ô*_*k*_ ∈ {*â*_*i*α_, *â*_*i*β_^†^}. The total spin of *Ô*, *S* ^op^, is not necessarily well-defined, and the operator has in general several spin components. These components range from a minimum spin of |*M* ^op^| to a maximum of $\frac{1}{2}N^{\mathrm{op}}$ .

A matrix element of the form ⟨*S*′, *M*′|*S* ^op^, *M* ^op^|*S*, *M*⟩ can only be nonzero if the coupling between two of the terms includes a component matching the third. In particular, the following must be satisfied

7 $$M^{\prime }=M+M^{\mathrm{op}}$$

8 $$\left|S-S^{\prime }\right|\leq S^{\mathrm{op}}\leq S+S^{\prime }$$

For a spin-adapted operator (with well-defined *S* ^op^), this is explicit enough, but otherwise it only means that *some* of the spin components of *Ô* must be between |*S* – *S*′| and *S* + *S*′. In this case, this latter condition can be replaced with

9 $$\begin{array}{l} \left|S-S^{\prime }\right|\leq \frac{1}{2}N^{\mathrm{op}}\\ S+S^{\prime }\geq \left|M^{\mathrm{op}}\right| \end{array}$$

For genealogical CSFs, this applies to any intermediate level *i*, i.e., to the CSFs and operator sequence obtained by considering only the first *i* elements of ***t*** and the elementary operators with level indices between 1 and *i*. Again, using the double values *b* and *x* for 2*S* and 2*M*, this translates to

10 $$\begin{array}{l} \left|\Delta b_{i}\right|\leq N_{i}^{\mathrm{op}}\\ b_{i}+b_{i}^{\prime }\geq \left|x_{i}^{\mathrm{op}}\right| \end{array}$$

where it is understood that Δ*b*_*i*_ = *b*_*i*_ – *b*_*i*_^′^, *N*_*i*_^op^ is the number of elementary operators at level *i* or lower, and *x*_*i*_^op^ is the *x* value computed only from those operators. If the operator is spin-adapted up to level *i*, it will have a well-defined *b*_*i*_^op^ value and the condition is then simplified to

11 $$\left|\Delta b_{i}\right|\leq b_{i}^{\mathrm{op}}\leq b_{i}+b_{i}^{\prime }$$

Specifically, for a singlet operator, constructed from any number of elementary operators, *b*_*i*_^op^ = 0, and this reduces to *b*_*i*_^′^ = *b*_*i*_.

An implicit condition in eq 8 is that the parity of 2*S*′ must be the same as that of 2*S* + 2*S* ^op^. This is enforced by [Disp-formula eq7], as the parities of 2*M* and 2*S* are necessarily equal. For intermediate CSF levels, *M* is not well-defined, unlike *S*, and this implicit condition must be explicitly expressed. Therefore, this can be added

12 $$\left|\Delta b_{i}\right|\equiv N_{i}^{\mathrm{op}}\left(\mathrm{mod}2\right)$$

ensuring that Δ*b*_*i*_ ∈ {0, ± 2, ± 4,...} for even *N*_*i*_^op^ or Δ*b*_*i*_ ∈ { ± 1, ± 3,...} for odd *N*_*i*_^op^. Any matrix element between genealogical CSFs not satisfying eq 7, and eqs 10 and 12 for every level *i*, is zero and does not need to be evaluated.

## The Algorithm

The calculation of ⟨ ***t*** ′, *M*′|*Ô*| ***t***, *M*⟩ is divided in two parts: first obtain *Ô* | ***t***, *M*⟩, and then its overlap with ⟨ ***t*** ′, *M*′|.

### Applying an Operator

It will be convenient to store, for a CSF | ***t***, *M*⟩, not only the ***t*** and *M* values, but also a vector ***b*** with all the *b*_*i*_ values and another vector ***r*** containing the cumulative number of singly occupied orbitals: *r*_*i*_ = ∑_*k* = 1_^*i*^(occ­(*t*_*k*_) mod 2). Thus, the following is stored for a CSF:

- ***t***: Vector of step values, *t*_*i*_ ∈ {0, u, d, 2}.
- ***b***: Vector of intermediate double spin values, *b*_*i*_ ∈ {0, 1, 2,...}.
- ***r***: Vector of cumulative singly occupied orbitals, *r*_*i*_ ∈ {0, 1, 2,...}.
- *M*: Final spin projection of the CSF, $\left|M\right|\leq \frac{1}{2}b_{n_{o}}$ .

As indicated above, applying an operator sequence *Ô* to a CSF results in a different GDG that is in general no longer a CSF and cannot be represented simply as | ***t***, *M*⟩. Nevertheless, it is not necessary to explicitly specify all the determinants in the linear combination. Such a GDG resulting from a modified CSF can be represented by adding some data to what is already stored for a CSF:

- ***q***: Vector of modified step values, *q*_*i*_ ∈ {0, u, d, α, β, 2}. For a CSF, ***q*** = ***t.***
- **τ**: Vector of original spin steps, τ_*i*_ ∈ {0, α, β}. For a CSF, **τ** = 0.
- ***d***: Vector of shifts, *d*_*i*_ ∈ {0, ± 1, ± 2,...}. For a CSF, ***d*** = 0.
- ϕ: Phase factor, ϕ ∈ { ± 1, 0}. For a CSF, ϕ = 1.

Then the effect of an elementary operator on this set is determined as detailed in Table 4. The first two columns of each subtable specify the operator and the current value of *q*_*i*_. The last four columns specify how ***q***, **τ**, ϕ and ***d*** should be updated. For *q*_*i*_ and τ_*i*_, they are simply set to the given value (when empty, no change is needed). For ϕ, it is either set to 0 or multiplied by ± 1 depending on the number of electrons up to level *i*: *N*_*i*_ = ∑_*k* = 1_^*i*^occ­(*q*_*k*_) (before updating *q*_*i*_). For ***d***, all elements at and above level *i* are added ± 1. The values of ***t***, ***b***, ***r*** and *M* are never modified, they correspond always to the original CSF.

**Table 4:**
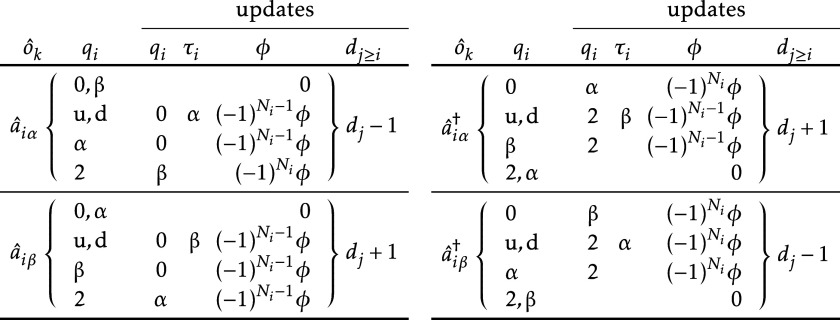
Effect of an Elementary Operator on the Data Stored for a GDG^a^

a For each operator and *q_i_* value, the new values for *q_i_*, *τ_i_*, ϕ and *d_j_* (*j* ≥ *i*) are listed.

Coming back to the previous example, the CSF |uud0uu2d, *M* = 1⟩ would be represented by

$$\begin{array}{l} t=(u,u,d,0,u,u,2,d),\;b=(1,2,1,1,2,3,3,2),\;r=(1,2,3,3,4,5,5,6),\;M=1\\ q=(u,u,d,0,u,u,2,d),\;\tau =(0,0,0,0,0,0,0,0),\;d=(0,0,0,0,0,0,0,0),\;\phi =1 \end{array}$$

Applying *â*_7β_, with *q*_7_ = 2, results in

q=(u,u,d,0,u,u,α,d),τ=(0,0,0,0,0,0,0,0),d=(0,0,0,0,0,0,1,1),ϕ=1

and then appyling *â*_1β_^†^, with *q*_1_ = u

q=(2,u,d,0,u,u,α,d),τ=(α,0,0,0,0,0,0,0),d=(−1,−1,−1,−1,−1,−1,0,0),ϕ=−1

### Building a Graph

From this set of quantities it would be possible to build a GDG. This is not necessary for computing the matrix element, but we detail the process here in order to clarify the following step. For an unmodified CSF, at each level the graph contains *b*_*i*_ + 1 nodes, with *x* coordinates in the range {−*b*_*i*_, −*b*_*i*_ + 2,..., *b*_*i*_–2, *b*_*i*_}. At the final level *n*_o_, the node corresponding to *M* has an *x* coordinate equal to 2*M*, and not all nodes at previous levels can have an influence on it, specifically only those at *x* coordinates between 2*M* – (*r*_*n*_o__ – *r*_*i*_) and 2*M* + (*r*_*n*_o__ – *r*_*i*_) are relevant. The range of *x* coordinates at each level can then be reduced to [*x*_*i*_^min^, *x*_*i*_^max^], with

13 $$\begin{array}{l} x_{i}^{\min}=\max\left(-b_{i},2M-\left(r_{n_{o}}-r_{i}\right)\right)\\ x_{i}^{\max}=\min\left(b_{i},2M+\left(r_{n_{o}}-r_{i}\right)\right) \end{array}$$

When a CSF is modified, its *x* coordinates are shifted by *d*_*i*_ at each level, and the modified step values could make some of the nodes unreachable. The *x* range for each level can be obtained from the range at the previous level, assuming *x*_0_^min^ = *x*_0_^max^ = 0

14 $$\begin{array}{l} x_{i}^{\min}=\max\left[x_{i-1}^{\min}+\delta_{i}^{\min},\max\left(-b_{i},2M-\left(r_{n_{o}}-r_{i}\right)\right)+d_{i}\right]\\ x_{i}^{\max}=\min\left[x_{i-1}^{\max}+\delta_{i}^{\max},\min\left(b_{i},2M+\left(r_{n_{o}}-r_{i}\right)\right)+d_{i}\right] \end{array}$$

where δ_*i*_^min^ and δ_*i*_^max^ are given in Table 5.

**Table 5:** Changes to the Allowed *x* Range Depending on the Step Value

*q*_*i*_	δ_*i*_^min^	δ_*i*_^max^
0, 2	0	0
u, d	–1	+1
α	+1	+1
β	–1	–1

With the data from the example we would get

$$x^{\min}=(0,-1,-2,-2,-1,0,1,2),\;x^{\max}=\left(0,1,0,0,1,2,3,2\right)$$

matching the ranges of reachable nodes in the left graph of Figure 2.

From each node (*x*, *i* – 1), there could be edges to nodes (*x* – 1,*i*), (*x*, *i*), or (*x* + 1, *i*), with weights given by the original CSF. Note that all phase factors are collected in ϕ, instead of modifying the edge weights. Table 6 provides the explicit values, based on the stored data.

**Table 6:** Edge Weights for Modified CSFs^a^

*q*_*i*_	(*x* – 1, *i*)	(*x*, *i*)	(*x* + 1,*i*)
0, 2	—	*C*_*t*_*i*_,τ̃_*i*__(*b*_*i*_, *x* – *d*_*i*_)	—
α	—	—	*C*_*t*_*i*_,τ_*i*__(*b*_*i*_, *x* – *d*_*i*_)
β	*C*_*t*_*i*_,τ_*i*__(*b*_*i*_, *x* – *d*_*i*_)	—	—
u,d	*C*_*t*_*i*_,β_(*b*_*i*_, *x* – *d*_*i*_)	—	*C*_*t*_*i*_,α_(*b*_*i*_, *x* – *d*_*i*_)

a The origin of the edge is always (*x*, *i* – 1). *τ̃_i_* is *τ*_i_ if *τ*_i_ ≠ 0, otherwise it is *t_i_*.

### Computing the Overlap

The overlap between two GDGs, given the stored quantities, can be computed from their reconstructed graphs, multiplied by the phase factors ϕ and ϕ′. Since the overlap between two graphs requires matching edges at every level to be nonzero, the following conditions can be stated, in addition to the spin selection rules detailed above

15 $$\mathrm{occ}\left(q_{i}^{\prime }\right)=\mathrm{occ}\left(q_{i}\right),\forall i$$

16 $$q_{i}^{\prime }\in \{q_{i},u,d\},\mathrm{if}{\;q}_{i}\in \left\{\alpha ,\beta \right\}$$

17 $$\phi^{\prime },\phi \neq 0$$

The condition [Disp-formula eq16] simply ensures that *q*_*i*_^′^/*q*_*i*_ are not α/β or β/α. To compute the overlap, we set a vector ***v*** to hold the node values at a particular level *i* (only one level at a time). The indices of ***v*** can be negative, as they refer to the *x* values, and they are always limited to the range [−*n*_o_, *n*_o_]. Initially all elements of ***v*** are set to 0, except *v*_0_ = ϕϕ′. Then, for each level successively, from *i* = 1 to *i* = *n*_o_, the values of ***v*** are updated. First the ranges [*x*_*i*_^min^, *x*_*i*_^max^] and [*x*_*i*_^′min^, *x*_*i*_^′max^] are obtained. Only the ***v*** values between max­(*x*_*i*_^min^, *x*_*i*_^′min^) and min­(*x*_*i*_^max^, *x*_*i*_^′max^), in a stride of 2, need to be updated, according to Table 7. All other *v*_*x*_ elements not in the updated range are set to 0. When *i* = *n*_o_ is reached, only one ***v*** value is updated, corresponding to *x* = 2*M* + *d*_*n*_o__ = 2*M*′ + *d*_*n*_o__^′^, and the final result for the overlap is *v*_*x*_. Note that for computing ⟨ ***t*** ′, *M*′|*Ô*| ***t***, *M*⟩ only the first and last three rows are needed, as *q*_*i*_^′^ corresponds to an unmodified CSF and will never be α or β.

**Table 7:** Updated Node Values for Computing the Overlap between Two GDGs^a^

*q*_*i*_	*q*_*i*_^′^	*v*_*x*_
0, 2	0, 2	*v*_*x*_ *C*_*t*_*i*_,τ̃_*i*__(*b*_*i*_, *x* – *d*_*i*_)*C*_*t*_*i*_^′^,τ̃_*i*_^′^_(*b*_*i*_^′^, *x* – *d*_*i*_^′^)
α	α	*v*_*x*–1_ *C*_*t*_*i*_,τ_*i*__(*b*_*i*_, *x* – *d*_*i*_) *C*_*t*_*i*_^′^,τ_*i*_^′^_(*b*_*i*_^′^, *x* – *d*_*i*_^′^)
β	β	*v*_*x*+1_ *C*_*t*_*i*_,τ_*i*__(*b*_*i*_, *x* – *d*_*i*_) *C*_*t*_*i*_^′^,τ_*i*_^′^_(*b*_*i*_^′^, *x* – *d*_*i*_^′^)
u, d	α	*v*_*x*–1_ *C*_*t*_*i*_,α_(*b*_*i*_, *x* – *d*_*i*_) *C*_*t*_*i*_^′^,τ_*i*_^′^_(*b*_*i*_^′^, *x* – *d*_*i*_^′^)
u, d	β	*v*_*x*+1_ *C*_*t*_*i*_,β_(*b*_*i*_, *x* – *d*_*i*_) *C*_*t*_*i*_^′^,τ_*i*_^′^_(*b*_*i*_^′^, *x* – *d*_*i*_^′^)
α	u, d	*v*_*x*–1_ *C*_*t*_*i*_,τ_*i*__(*b*_*i*_, *x* – *d*_*i*_) *C*_*t*_*i*_^′^,α_(*b*_*i*_^′^, *x* – *d*_*i*_^′^)
β	u,d	*v*_*x*+1_ *C*_*t*_*i*_,τ_*i*__(*b*_*i*_, *x* – *d*_*i*_) *C*_*t*_*i*_^′^,β_(*b*_*i*_^′^, *x* – *d*_*i*_^′^)
u, d	u, d	*v*_*x*–1_ *C*_*t*_*i*_,α_(*b*_*i*_, *x* – *d*_*i*_) *C*_*t*_*i*_^′^,α_(*b*_*i*_^′^, *x* – *d*_*i*_^′^) + *v*_*x*+1_ *C*_*t*_*i*_,β_(*b*_*i*_, *x* – *d*_*i*_) *C*_*t*_*i*_^′^,β_(*b*_*i*_^′^, *x* – *d*_*i*_^′^)

a *τ̃_i_* is τ_*i*_ if *τ_i_* ≠ 0, otherwise it is *t_i_*.

If κ is the lowest elementary operator in *Ô* (κ = min_*k*_{*i*_*k*_}), then for the levels below, *i* < κ, it will always be the case that *q*_*i*_ = *t*_*i*_, *q*_*i*_^′^ = *t*_*i*_^′^, *d*_*i*_ = *d*_*i*_^′^ = 0, and due to the orthonormality of the CSFs, the node values at level *i* will all be 0 if *t*_*i*_^′^ ≠ *t*_*i*_ or equal to the values at level *i* – 1 if *t*_*i*_^′^ = *t*_*i*_. Therefore, the overlap can be computed starting from level κ, setting the initial ***v*** as ϕϕ′ for elements *x* ∈ {−*b*_min_, −*b*_min_ + 2,..., *b*_min_ – 2, *b*_min_} where *b*_min_ = *b*_*κ*–1_.

It is instructive to compare the complexity of this algorithm with a straightforward expansion into Slater determinants. Let us consider CSFs of the type |uu···ud···dd⟩, with *S* = 0 and an even number, *n*, of orbitals and electrons. The number of Slater determinants in the expansion scales exponentially with *n*, while the number of nodes and edges in the corresponding graph only grows quadratically

18 $$N_{\mathrm{SD}}=\left(\begin{array}{c} n\\ n/2 \end{array}\right)$$

19 $$N_{\mathrm{node}}={\left(n/2+1\right)}^{2}{,N}_{\mathrm{edge}}=n\left(n/2+1\right)$$

The proposed algorithm would require a number of operations proportional to the number of nodes, and therefore it is expected to be more favorable as the number of orbitals increases. For example, for *n* = 20, *N*_SD_ = 184756 and *n*_node_ = 121.

### Summary

To summarize, the algorithm can be stated in the following steps:

1. From ***t*** and ***t*** ′ obtain ***b***, ***b*** ′, ***r***, ***r*** ′. The auxiliary quantities ***q***, ***q*** ′, **τ**, **τ**′, ***d***, ***d*** ′, ϕ, ϕ′ are set to defaults.
2. Ensure eq 7 is satisfied, as well as eqs 10 and 12 for every level *i*. If not, the result is 0.
3. Apply *Ô* | ***t***, *M*⟩ to update ***q***, **τ**, ***d***, ϕ (Table 4). If at any point ϕ = 0, the result is 0.
4. Ensure eqs 15 and 16 are satisfied. If not, the result is 0.
5. Find the lowest index in the operator, κ, and the *b* value at level κ – 1, *b*_min_. Set *v*_*x*_ = *ϕϕ*′ for *x* ∈ {−*b*_min_,–*b*_min_ + 2,..., *b*_min_ – 2,*b*_min_} and *v*_*x*_ = 0 otherwise.
6. Update the elements of ***v***, from *i* = κ to *i* = *n*_o_ (Table 7). If at any step no elements of ***v*** are updated, or if all the updated elements are 0, the result is 0.
7. The final result is *v*_*x*_, the single element updated in the last step.

## Numerical Tests

As a proof of concept, we have implemented the suggested algorithm in a Python script, and we compare it to a simple algorithm based on an explicit determinant expansion of the CSFs. We select a number of random CSFs given a fixed number of electrons (*N*), number of orbitals (*n*_o_), spin (*S*), and spin projection (*M*); for each of these CSFs an operator sequence of *N* ^op^ elementary operators (half of them creation, half annihilation) is generated, by randomly selecting an occupied orbital and spin for each annihilation operator and a not doubly occupied orbital and spin for each creation operator, and another random CSF is chosen from those that could have a nonzero coupling with the first CSF, according to the spin and occupation selection rules. This gives a random list of *n*_coup_ matrix elements for each set. These matrix elements are computed with both algorithms and the results and timings are compared.

In Table 8 the results are collected. We have tested CSFs up to 34 electrons in 34 orbitals (close to the limit where the number of CSFs does not fit in a standard 64-bit integer), with sequences of 2 or 8 elementary operators (relevant for one- and four-particle density matrices). For each matrix element, the reference value is computed in symbolic form with the graph-based algorithm. Against this reference the numerical results of both algorithms are compared, and the accuracy is measured in terms of root-mean-square deviation and maximum absolute error. From the timings in the table it is clear that the graph-based algorithm is much faster than explicit determinant expansion, and the benefit increases as the number of orbitals and electrons (and therefore the number of determinants per CSF) gets larger. In general, the timing of the determinant-based algorithm is roughly proportional to the number of determinants involved, while the timing of the graph-based algorithm is more or less proportional to the number of matrix elements to compute. The accuracy of the graph-based algorithm is close to machine precision in all cases, but the determinant-based algorithm can introduce somewhat larger numerical errors. It is significant that the errors are always larger for *N* ^op^ = 2 than for *N* ^op^ = 8, this is probably because the number of actual contributions to the final result in the latter case is much smaller with both algorithms, greatly reducing accumulated rounding errors. For *N* ^op^ = 2, the number of determinants contributing per matrix element grows exponentially with *N* and *n*_o_, and so do the errors of the determinant-based algorithm.

**Table 8:** Results over Random Lists of *n*_coup_ Matrix Elements between CSFs, ⟨ ***t*** ′, *M*′|*Ô*| ***t***, *M*⟩^a^

				determinants			graph		
(*N*, *n*_o_, *S*)	*n*_coup_	*N* ^op^	*n*_det_/10^6^	time/s	rmsd/10^–16^	max/10^–16^	time/s	rmsd/10^–16^	max/10^–16^
$\left(15,15,\frac{1}{2}\right)$	10^6^	2	45.76	1496	1.22	82.2	246.5	0.61	4.44
		8	46.46	1412	0.23	6.7	324.4	0.21	3.33
(16, 16, 1)	10^6^	2	87.85	2895	1.28	94.4	267.7	0.64	5.55
		8	88.95	2457	0.25	5.0	312.8	0.23	0.23
(20, 20, 0)	10^5^	2	26.41	1069	3.13	499.6	32.6	0.66	5.55
		8	26.80	885	0.17	4.2	37.0	0.16	2.22
$\left(21,21,\frac{5}{2}\right)$	10^5^	2	80.62	3495	5.15	369.7	38.8	0.43	4.44
		8	54.00	1921	0.12	3.9	40.8	0.09	2.22
$\left(25,25,\frac{1}{2}\right)$	10^4^	2	14.21	746	7.77	254.8	4.1	0.41	4.44
		8	14.58	607	0.10	2.5	4.9	0.08	1.67
(28, 28, 0)	10^4^	2	45.33	2532	26.94	814.9	4.6	0.48	3.33
		8	43.03	1935	0.12	5.3	5.3	0.07	0.07
(34, 34, 2)	10^3^	2	33.93	2191	71.67	2059.0	0.6	0.23	2.22
		8	34.68	1944	0.10	1.9	0.7	0.03	0.28
$\left(35,35,\frac{3}{2}\right)$	10^3^	2	38.38	2539	91.51	2418.0	0.6	0.26	3.33
		8	105.33	5926	0.08	1.9	0.7	0.02	0.28

a *N*, *n*_o_, and *S* are respectively the number of electrons, number of orbitals and spin of | ***t***, *M*⟩, *N* ^op^ is number of elementary operators in *Ô*, *n*_det_ is the total number of determinants in the expansion of all | ***t***, *M*⟩ (including possible duplicates). The matrix elements are computed with both a determinant-based algorithm and the graph-based algorithm proposed here; for each, “time” is the total time spent, “rmsd” is the root mean square deviation of the result compared to the symbolic result, “max” is the maximum absolute error.

The scaling of the two algorithms is compared in Figure 3. For the determinant-based algorithm we plot the time per determinant, observing that it increases with the number of orbitals. This is expected, as the cost of storing and processing a single determinant should scale linearly with the number of orbitals. For the graph-based algorithm we represent the time per matrix element, and the trend is also linear. The fact that it is not quadratic, as would be expected from the graph sizes (see eq 18), may indicate that in this range the cost is still dominated by processing the CSF data vectors and not by the computation of the ***v*** values. It is interesting to note that the timings seem to increase with the number of operators for the graph-based algorithm, but the opposite is found for the determinant-based algorithm. To explain this, we point out that as the number of operators increases, it becomes more likely that a single determinant will be annihilated, and annihilated determinants hardly contribute to the total timing. In the graph-based algorithm increasing the number of operators would similarly reduce the graph size, but in this case the overhead of processing the effect of the operators probably offsets the possible gain and makes the final timing larger.

**Figure 3:**
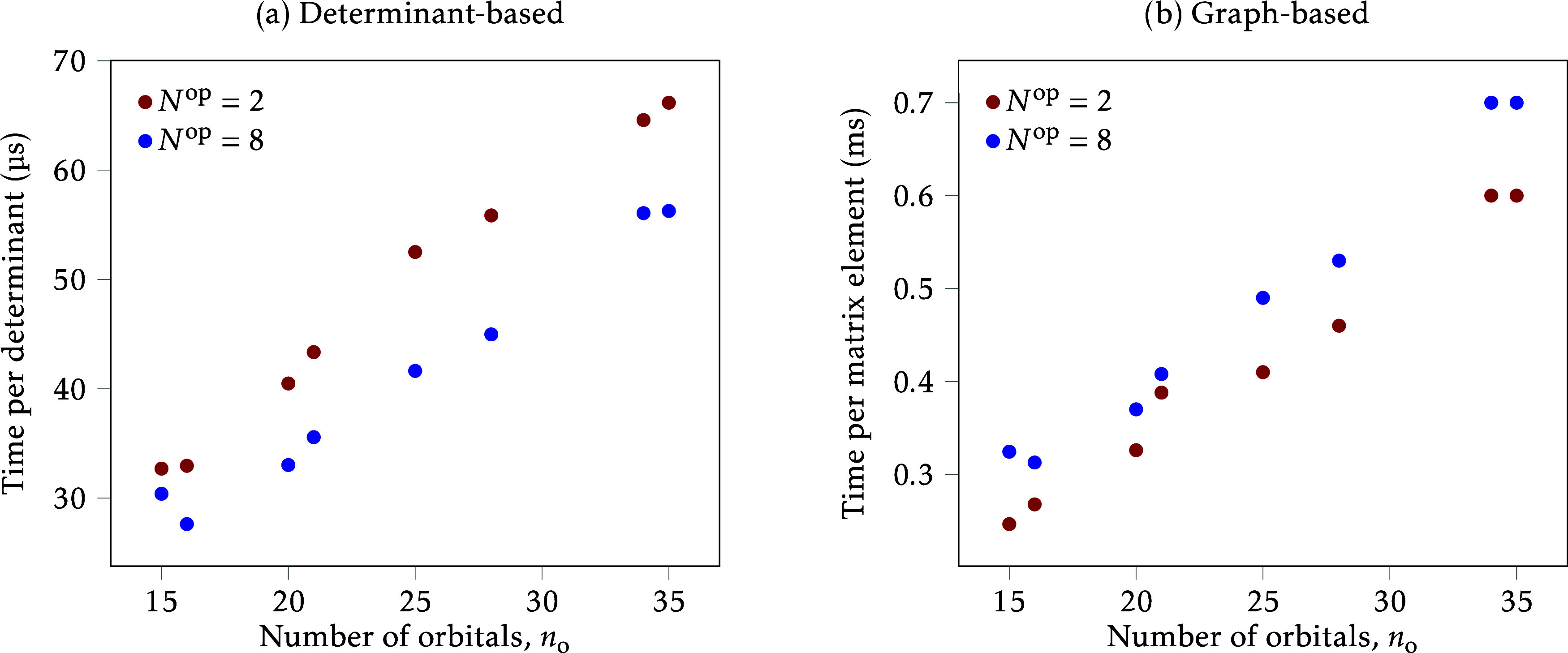
Timing plots for the two algorithms. (a) Determinant-based algorithm, total time is divided by the number of determinants in the ket CSF (*n*_det_). (b) Graph-based algorithm, total time is divided by the number of matrix elements computed (*n*_coup_).

These results show the good behavior of the proposed algorithm. However, although we compare the timings, we do not claim this should be the preferred algorithm. We have only compared the calculation of individual matrix elements between CSFs, but in an actual application one is more concerned with computing the density matrices between wave functions consisting of linear combinations of CSFs, and for this task more efficient algorithms should be used. For example, it is probably advantageous to transform the set of CSFs to a common set of determinants,^3,26^ instead of doing the expansion for each CSF separately, and more compact and efficient algorithms exist for spin-adapted operators.^11,15,27^ Moreover, no particular effort has been taken to optimize either of the implementations used in this comparison. Nevertheless, we believe this algorithm provides a basis for further developments that can be especially useful in the context of selected^28,29^ or stochastic CI^30,31^ methods with large active spaces, where any expansion in Slater determinants would be a hindrance.

## Conclusion

An algorithm has been proposed to compute matrix elements of arbitrary operators between genealogical CSFs. The algorithm is based on a graphical representation of the Slater determinants contributing to the CSFs, but does not require either building the graph or expanding the CSFs into determinants. Compared with a naive determinant expansion, the performance can improve by several orders of magnitude. Although other algorithms exist that exhibit better performance and scaling properties, the one proposed in this work offers the ability to treat arbitrary operators expressed in terms of second-quantization creation and annihilation operators. In addition, the graphical algorithm is conceptually simple and with didactic potential, it can be easily carried out with pen and paper. We suggest this approach can be of use for methods that do not have a fixed list of configurations (determinants or CSFs) contributing to the wave function, such as selected CI or stochastic CI.

## Supplementary Material





## Notes

Python codes implementing the proposed algorithm and a plain determinant-based approach (PDF)
Lists of matrix elements computed in the numerical tests (ZIP)
