# Supplementary material for: A Graph-Based Algorithm for Computing Matrix Elements of Arbitrary Operators between Configuration State Functions
Source: J Phys Chem A. 2026 Feb 18;130(9):1924–32. doi: 10.1021/acs.jpca.5c08310 (PMC12969370; doi:10.1021/acs.jpca.5c08310)
Supplement: Supplementary file 1 [file jp5c08310_si_001.pdf]

Supporting Information: A Graph-Based Algorithm for Computing Matrix Elements of Arbitrary Operators between Configuration State Functions

Ignacio Fdez. Galván,*,† Mitra Rooein,*,† and Roland Lindh*,†,‡

†*Department of Chemistry for Life Sciences, Uppsala University, P. O. Box 576, 75123 Uppsala, Sweden*

‡*Uppsala Center for Computational Chemistry (UC_3), Uppsala University, P. O. Box 576, 75123 Uppsala, Sweden*

E-mail: ignacio.fernandez@kemi.uu.se; mitra.rooein@kemi.uu.se; roland.lindh@kemi.uu.se

Contents of the `SI.zip` file:

- `README.txt`:

  This description.

- `list_N,P,Y_X.txt`:

  The files `list_N,P,Y_X.txt` contain lists of matrix elements between two CSFs ($\langle m'|\hat{O}|m\rangle$) such that m has N electrons, P orbitals, and spin multiplicity Y, and where $\hat{O}$ is a sequence of X elementary operators ($X/2$ creation, $X/2$ annihilation). The first line in each file gives the total number of CSFs consistent with the N, P, Y constraints, then each line gives a matrix element with the format:

  ```
  <t',M'|o1 ... oX|t,M> = C (n)
  ```

  `t`, `t'`: step vectors identifying each CSF (0: empty, u: 1 electron increasing spin, d: 1 electron decreasing spin, 2: doubly occupied)

  `M`, `M'`: spin projection value for each CSF

  `o1 ... oX`: sequence of operators (each operator is: [a: annihilation, c: creation] orbital index [a: alpha, b: beta])

  `C`: value of the matrix element in symbolic form

  `n`: number of Slater determinants in the expansion of the ket CSF (m).

- `graph_based.py`, `determinant_based.py`:

  The scripts `graph_based.py` and `determinant_based.py` compute the matrix elements with two different methods, they can be imported in other Python scripts, e.g.:

```
import graph_based

# Set symbolic computing to True or False
graph_based.set_symbolic(True)

bra_str = '2uu0duud'
bra_M = 1
ket_str = 'uud0uu2d'
ket_M = 1
op_str = 'c1b a7b'
value = graph_based.coupling(bra_str, bra_M, op_str, ket_str, ket_M)
print(f'<{bra_str},{bra_M}|{op_str}|{ket_str},{ket_M}> = {value})
```

or they can be used from the command line, specifying the matrix element in the format above (it will probably require quoting), e.g.:

```
$ ./graph_based.py '<2uu0duud,1|c1b a7b|uud0uu2d,1>'
<2uu0duud,1.0|c1b a7b|uud0uu2d,1.0> = -0.1527777777777778
```

In this form, they admit the flag “-s” to enable symbolic calculation:

```
$ ./graph_based.py -s '<2uu0duud,1|c1b a7b|uud0uu2d,1>'
<2uu0duud,1.0|c1b a7b|uud0uu2d,1.0> = -11/72
```
